# CD11c^−^MHC2^low^ Macrophages Are a New Inflammatory and Dynamic Subset in Murine Adipose Tissue

**DOI:** 10.20900/immunometab20200015

**Published:** 2020-04-17

**Authors:** Suzan Wetzels, Mitchell Bijnen, Erwin Wijnands, José van de Gaar, Andika Tan, Susan Coort, Erik A. L. Biessen, Casper G. Schalkwijk, Kristiaan Wouters

**Affiliations:** 1 Department of Pathology, Cardiovascular Research Institute Maastricht (CARIM), Maastricht University, Maastricht 6229 ER, the Netherlands; 2 Department of Stromal Cell Immunobiology, Aix Marseille Univ, CNRS, INSERM, CIML, Marseille 13009, France; 3 Central Diagnostic Laboratory, Maastricht Universitair Medisch Centrum MUMC+, 6229 HX, the Netherlands; 4 Department of Internal Medicine, Cardiovascular Research Institute Maastricht (CARIM), Maastricht University, Maastricht 6229 ER, the Netherlands; 5 Department of Bioinformatics-BiGCaT, School for Nutrition and Translational Research in Metabolism (NUTRIM), Maastricht University, Maastricht 6229 ER, the Netherlands

**Keywords:** adipose tissue macrophages, MHC2, CD11c, visceral adipose tissue, flow cytometry, bone marrow transplantation, free fatty acids

## Abstract

**Background:**

The prevalence of obesity is rising and leads to increased morbidity and mortality. Adipose tissue inflammation, due to accumulation and activation of adipose tissue macrophages (ATMs), is a key driver of this phenomenon. Macrophages are heterogeneous cells, adapting quickly to the microenvironment, resulting in so-called M1 or M2 macrophages. In this study, we describe the dynamics and inflammatory properties of a newly identified ATM subset in obese mice.

**Methods:**

LDLR^-/-^ mice received a high fat diet (HFD) for 5 weeks or 16 weeks to induce obesity. Adipose tissues were isolated and immune cell subsets were analyzed with flow cytometry or microarray analysis. Bone marrow transplantation (BMT) using CD45.1 and CD45.2 LDLR^-/-^ mice was performed to determine ATM origin.

**Results:**

Upon HFD, there is a massive increase of ATM subsets in the adipose tissue. CD11c^−^M2 ATMs could be subdivided based on their MHC2 expression into CD11c^−^MHC2^high^ ATMs and previously unidentified CD11c^−^MHC2^low^ ATMs. CD11c^−^MHC2^low^ ATMs accumulated very rapidly after 10 days of HFD, after which they increased even further with prolonged HFD. Microarray data showed that CD11c^−^MHC2^low^ ATMs resembled CD11c^−^MHC2^high^ ATMs in the steady state, but became more inflammatory during development of obesity. In vitro stimulation of bone marrow-derived macrophages with palmitate, abundantly present in HFD, resulted in the induction of the CD11c^−^MHC2^low^ phenotype.

**Conclusions:**

Among M2 macrophages, a novel pro-inflammatory subset of macrophages was found based on their low level of MHC2 expression. This subset may play a role in the development of adipose tissue inflammation.

## Introduction

The ever-rising prevalence of obesity represents one of the major problems in modern health care and healthy aging. Obesity predisposes to “cardiometabolic” diseases like type 2 diabetes (T2D) [1], nonalcoholic fatty liver disease (NAFLD) [2], and to atherosclerotic plaque formation and vascular dysfunction leading to cardiovascular disease (CVD) [3]. Together, these conditions account for the majority of morbidity and mortality in the world. It is now widely accepted that inflammation induced by adipose tissue macrophages (ATMs) is a key driver of these conditions [4–7]. Indeed, accumulation of ATMs is causally linked to the development of insulin resistance leading to impaired glucose homeostasis and T2D, in turn contributing to hepatic and vascular complications [6]. Moreover, ATMs directly cause immune cell recruitment from the bone marrow leading to inflammation in the liver and worsening NAFLD, irrespective of insulin resistance [8]. Thus, adipose tissue inflammation and ATM accumulation are central drivers of cardiometabolic diseases. Despite the existing experimental knowledge about the role of adipose tissue inflammation, targeting the inflammatory component of obesity in humans remains unsuccessful [4].

Macrophages display a high degree of plasticity depending on stimuli from their environment. Classically activated (M1) ATMs are pro-inflammatory and accumulate in expanding adipose tissue, causing inflammation [6]. On the other end of the spectrum, alternatively activated (M2) ATMs are anti-inflammatory and protect against the development of cardiometabolic diseases [6]. The presence of CD11c on the surface of human and mouse ATMs is discriminative for M1 ATMs and the accumulation of CD11c^+^ ATMs correlates with insulin resistance [9]. The M1/M2 dogma relies mainly on in vitro data and scientists start to appreciate that this subdivision is a vast oversimplification of tissue macrophage diversity in vivo [5]. The microenvironment of obese adipose tissue is different than lean adipose tissue. It is known that the expression of cytokines and chemokines is increased in hypertrophied adipocytes. Moreover, free fatty acids (FFAs) are increased in the microenvironment of the obese adipose tissue. Oleate (OA) is the most abundant FFA in human adipose tissue, whereas palmitate (PA) is the second most abundant [10]. Importantly, circulatory FFA levels are elevated in obesity [11,12]. This could imply that the microenvironment, FFAs in particular, have the potential to affect macrophage phenotype in vivo during obesity.

In this study, we assess the ATM subsets and their origin in LDLR^-/-^ mice after high fat diet (HFD). The LDLR^-/-^ mouse model lacks the low density lipoprotein (LDL) receptor which is responsible for the uptake of LDL from the circulation into tissue. Due to the knockout of LDLR, LDLR^-/-^ mice on HFD will develop obesity with elevated levels of lipids in their circulation making it an excellent model to study the metabolic syndrome. Here, we discovered a previously undescribed ATM subset: CD11c^−^MHC2^low^ ATMs. We unraveled the characteristics of this novel ATM subset and investigated the potential role of FFAs in shifting macrophages towards this subset.

## Materials and Methods

### Animal Studies

#### High fat diet

C57BL/6 LDLR^-/-^ mice (in house breeding colony, Maastricht University) were fed a high fat diet (HFD, 60% of kcal from fat, Research diets, New Brunswick USA) for 5 weeks (*n* = 7) or 16 weeks (*n* = 7) with their controls on a matched control diet (Research diets, New Brunswick USA) for respectively 5 (*n* = 7) or 16 weeks (*n* = 7). LDLR^-/-^ mice develop hyperlipidemia and due to the high fat content in the diet become obese and display insulin resistance [13], resulting in an excellent model to study the metabolic syndrome. After the HFD, the mice were sacrificed by CO_2_ inhalation and visceral adipose tissue (vAT) was isolated for flow cytometry analysis.

#### Clodronate liposome depletion of ATMs

C57BL/6 LDLR^-/-^ mice were fed a HFD for 16 weeks. Two days prior to sacrifice, the mice (*n* = 3) were intraperitoneally injected with 115 mg/kg clodronate liposomes (Clodronate liposomes.com, Amsterdam, the Netherlands). After sacrifice, the vAT was isolated for flow cytometry analysis.

#### Bone marrow transplantation

C57BL/6 LDLR^-/-^ CD45.2 acceptor mice (in house breeding colony, Maastricht University) were lethally irradiated with two doses of 6 Gy, 24 h apart. C57BL/6 LDLR^-/-^ CD45.1 donor mice (in house breeding colony, Maastricht University) were sacrificed by carbon dioxide inhalation followed by cervical dislocation. Subsequently, bone marrow was isolated from femur and tibia, pooled and dissolved in RPMI medium (Gibco^®^ 1640, Carlsbad, CA, USA) supplemented with heparin (Leo Pharma, Ballerup, Denmark) at a concentration of 50 × 10^6^ cells/mL. Ten million donor bone marrow cells were injected in the tail vein of each acceptor mouse. Acceptor mice received 100 mg/mL Neomycin (Gibco, Carlsbad, CA, USA) and 60 000 units/mL Polymyxin (p4932, Sigma-Aldrich, St. Louis, MO, USA) antibiotics in their drinking water 1 week before and 3 weeks after bone marrow transplantation. After 6 weeks recovery, mice were given either a HFD for 10 days (*n* = 6) or 5 weeks (*n* = 7), or a control diet for 5 weeks (*n* = 6). Subsequent to the diet, the mice were sacrificed by carbon dioxide inhalation followed by cardiac puncture and vAT was isolated for flow cytometry analysis.

#### Sorting of ATM subsets

C57BL/6 LDLR^-/-^ mice were fed a HFD for 16 weeks (*n* = 12) or a control diet (*n* = 18). Mice were sacrificed by CO_2_ inhalation, vAT was isolated for cell sorting using the flow cytometry staining panels. The ATMs were sorted in three groups: CD11c^+^ ATMs, CD11c^−^MHC2^high^ ATMs and CD11c^−^MHC2^low^ ATMs, using the FACS Aria (BD Biosciences, San Jose, CA, USA). ATMs from the vAT of *n* = 4 HFD mice were pooled and ATMs from the vAT of *n* = 6 control mice were pooled to create 3 pooled samples in order to gain sufficient amount of RNA to conduct the microarray analysis. The details of the amount of cells used for RNA extraction is presented in Supplemental Table S1.

#### OVA uptake by ATM subsets

C57BL/6 LDLR^-/-^ mice were fed a HFD for 5 weeks (*n* = 4) or a control diet (*n* = 4). One hour prior to sacrifice, the mice received an intraperitoneal injection with 2.5 μg DQ-OVA (Life Technologies, Carlsbad, CA, USA). Mice were sacrificed by CO_2_ inhalation and vAT was isolated to determine the DQ-OVA positive ATM subsets using flow cytometry. All performed procedures were approved by the Committee of Animal Welfare of Maastricht University (2012-20, date approved: 2/13/2012; 2012-164, date approved: 3/14/2012; 2013-092, date approved: 12/01/2013; 2014-059, date approved: 10/22/2014).

### Microarray

RNA from sorted ATMs was isolated and 3 ng total RNA was amplified using the Ovation PicoWTA system V2 (NuGEN, Leek, the Netherlands). Afterwards, SPIA cDNA was purified using Agencourt RNAClean XP beads (Beckman Coulter, Brea, CA, USA) and 2.8 mg was fragmented and labeled using the Encore Biotin module (NuGEN). All labeled samples were hybridized to Mouse Gene 2.1 ST arrays (Affymetrix, Santa Clara, CA, USA).

Washing, staining and scanning was performed using the GeneTitan Hybridization, Wash, and Stain Kit for WT Array Plates, and the GeneTitan Instrument (Affymetrix, Santa Clara, CA, USA). Data were normalized using GC-RMA normalization using Expression console software. Quality control was performed and all samples passed the quality control test. Next, data was analyzed using transcriptome analysis console v2.0 from Affymetrix using *t*-test from the R/Bioconductor package Limma. Data was corrected for multiple testing using false discovery rate (FDR). Gene ontology enrichment analysis was performed using The Database for Annotation, Visualization and Integrated Discovery (DAVID) v6.8 (https://david.ncifcrf.gov/). Pathway annotations were validated using WikiPathways (https://www.wikipathways.org/) and Reactome (https://reactome.org/). Data is presented as fold change (FC) in expression level compared to LFD fed mice. Genes showing fold changes of at least 1.50 and a *P*-value of less than 0.05 were considered differentially regulated.

### Cytokine Measurements

Cytokines were measured, as described previously [14], in the plasma of LDLR^-/-^ mice after 5 (*n* = 7) and 16 (*n* = 7) weeks of HFD using the V-plex multi-array electrochemiluminescense detection platform of MesoScaleDiscovery (MesoScaleDiscovery, Rockville, MD, USA) following manufacturer’s instruction.

### Culturing of Murine Bone Marrow-Derived Macrophages and Stimulation with Fatty Acids

C57BL/6J mice were sacrificed by cervical dislocation and femur and tibia were isolated. Femur and tibia were flushed with PBS through a 70 μM filter to obtain a single cell suspension. After centrifuging, the cells were resuspended in complete medium: RPMI 1640 GlutaMAX (Gibco, Carlsbad, CA, USA), supplemented with 1% glutamine penicillin/streptomycin, 10% heat-inactivated fetal calf serum (Greiner Bio-One, Kremsmünster, Austria) and 15% L929 cell conditioned medium (LCM). Cells were cultured for 7 days to allow differentiation into bone marrow-derived macrophages (BMDMs).

Fatty acids PA (5 mM, Sigma-Aldrich, Saint Louis, MO, USA) and OA (50 mM, Sigma-Aldrich, Saint Louis, MO, USA) were dissolved in 96% ethanol. Next, the solutions were saponified by adding NaOH at a 5:1 (PA:NaOH) or 1:2 (OA:NaOH) ratio. The saponified solutions were evaporated under N2 at 37 °C. Boiling MQ was added to resuspend the saponified fatty acids. The fatty acids were dissolved in RPMI 1640 (Gibco, Carlsbad, CA, USA) containing 1% BSA (Sigma-Aldrich), resulting in solutions of 500 μM PA and 500 μM OA. BMDMs were stimulated with 50 μM PA, 50 μM OA and a combination of 50 μM PA and 50 μM OA for 16 h. After stimulation, BMDMs were washed with PBS and harvested for RNA isolation using TriReagent (Sigma-Aldrich, Saint Louis, MO, USA) or flow cytometry.

### RNA Isolation and RT-qPCR

RNA from BMDMs was isolated using TriReagent (Sigma-Aldrich, Saint Louis, MO, USA) and cDNA was synthesized using the iScript™ cDNA Synthesis Kit (Bio-rad, Hercules, CA, USA) according to manufacturer’s instructions. RT-qPCR was performed using the 2X SensiMix™ SYBR® & Fluorescein Kit (Bioline, London, UK). The ΔΔCt method was used to determine the gene expression of the target genes CD11c (FW: CTGGATAGCCTTTCTTCTGCTG, RV: GCACACTGTGTCCGAACTCA) and MHC2 (FW: CCTGGTGACTGCCATTACCT, RV: GTAGCACTCGCCCATGAACT), relative to the house keeping genes Cyclophilin (FW: TTCCTCCTTTCACAGAATTATTCCA, RV: CCGCCAGTGCCATTATGG) and Beta-2 microglobulin (FW: CTTTCTGGTGCTTGTCTCACTGA, RV: GTATGTTCGGCTTCCCATTCTC). The CFX96 Touch™ Real-Time PCR Detection System (Bio-rad, Hercules, CA, USA) was used for thermal cycling and fluorescence detection.

### Flow Cytometry

vAT was incubated for 30 min at 37 °C with collagenase mix containing collagenase XI (1200 U/mL, Sigma-Aldrich, Saint Louis, MO, USA), collagenase I (3700 U/mL, Sigma-Aldrich, Saint Louis, MO, USA) and DNAse I (6760 U/mL, Sigma-Aldrich, Saint Louis, MO, USA) to digest the tissue. Samples were shaken every 5 min to improve the digestion. Subsequently, samples were filtered using 70 uM filters (2017500, BD Biosciences, CA, USA), centrifuged for 5 min at 1200 RPM and the floating adipocyte fraction was removed. The remaining pellet contained the stromal vascular fraction (SVF) and the erythrocytes were eliminated using a lysis buffer (pH = 7.3) containing NH4Cl (8.26 g/L), KHCO_3_ (1 g/L) and EDTA (0.037 g/L). After 1 min incubation with lysis buffer, samples were washed with PBS. Fc-blocking solution (16-01661, eBioscience, Vienna, Austria) containing CD16/CD32 antibodies was added to prevent nonspecific binding of antibodies and samples were incubated for 30 min. Subsequently, samples were stained with two antibody mixes for 30 min. Mix 1 consisted of CD45, CD45.1, CD45.2, NK1, CD11b, Ly6G, F4/80, CD3, CD19, CD11c and MHC2 (eBioscience, Vienna Austria), and was used to detect the different subpopulations of adipose tissue macrophages in the adipose tissue. The second mix consisted of CD45, CD45.1, CD45.2, CD3, NK1, Ly6G, CD11b, CD68, CD8 and CD4 (eBioscience, Vienna, Austria), and was used to detect granulocytes, monocytes, NK-cells and T-cells. Samples were washed twice with FACS buffer (10 mM NaN3, 0.5% BSA in PBS) and measured using FACS Canto II flow cytometer (BD Biosciences, San Jose, CA, USA). Data was analyzed using FACS Diva software version 6.12. The flow cytometry plots showing the gating strategy are depicted in Supplemental Figure S1. In short, CD45 staining was used to select the immune cells from singlet live cells in the adipose tissue. B-cells, T-cells, NK cells and granulocytes were excluded using B220, NK1, CD3 and Ly6G staining. Next, CD11b and F4/80 were used to select CD11c^−^ ATMs and CD11c^+^ cells. CD11b staining was used to subdivide the CD11c^+^ cells in CD11c^+^CD11b^high^ ATMs and CD11c^+^CD11b^low^ dendritic cells. CD11c^−^ cells were subdivided based on MHC2 expression in CD11c^−^MHC2^low^ and CD11c^−^MHC2^high^ ATMS.

vAT of mice injected with DQ-OVA was processed using collagenase mix and erythrocyte lysis buffer as described above. Cells were incubated with Fc blocking solution (16-01661, eBioscience, Vienna Austria) for 15 min. Next, cells were stained with CD45, NK, CD11b, Ly6G, Ly6C, CD3, CD19, CD11c, MHC2, MerTK and F4/80 (eBioscience, Vienna, Austria) for 30 min. Excess antibody was removed by washing twice with FACS buffer and samples were measured using FACS Canto II flow cytometer (BD Biosciences, San Jose, CA, USA). Data was analyzed using FACS Diva software version 6.12. Gating strategy was applied as described above (Supplemental Figure S1).

BMDMs stimulated with fatty acids were harvested using 10 mM EDTA-PBS. Fc-blocking was performed using CD16/CD32 antibodies (16-01661, eBioscience, Vienna, Austria) for 15 min. BMDMs were stained with F4/80, CD11c and MHC2 (eBioscience, Vienna, Austria) for 30 min. Excess antibody was removed by washing twice with FACS buffer and samples were measured using FACS Canto II flow cytometer (BD Biosciences, San Jose, CA, USA). Data was analyzed using FACS Diva software version 6.12. The following gating strategy was used: the singlet’s were determined using forward scatter-area (FSC-A) and FSC-height (FSC-H), and using the side scatter-width (SSC-W) and SSC-height (SSC-H). Next, the live fraction was determined based on FSC-A and SSC-area (SSC-A). The cells positive for F4/80 were gated and these were used to subdivide into CD11c^+^ and CD11c^−^ fraction of BMDMs. The CD11c^−^ fractions was used to determine the CD11c^−^MHC2^high^ and CD11c^−^MHC2^low^ BMDMs.

### Statistical Analysis

All data is presented as mean ± SEM. Data was analyzed with GraphPad Prism version 6 using Mann Whitney *t*-test and unpaired student *t*-test to compare two groups, and one-way ANOVA with Tukey’s multiple comparison test or Dunnett’s multiple comparison test to compare multiple groups. A *p* < 0.05 was considered significant.

## Results

### CD11c^−^MHC2^low^ Adipose Tissue Macrophages Are a Novel and Dynamic Subpopulation of Macrophages in the Adipose Tissue of LDLR^-/-^ Mice

**Figure 1:**
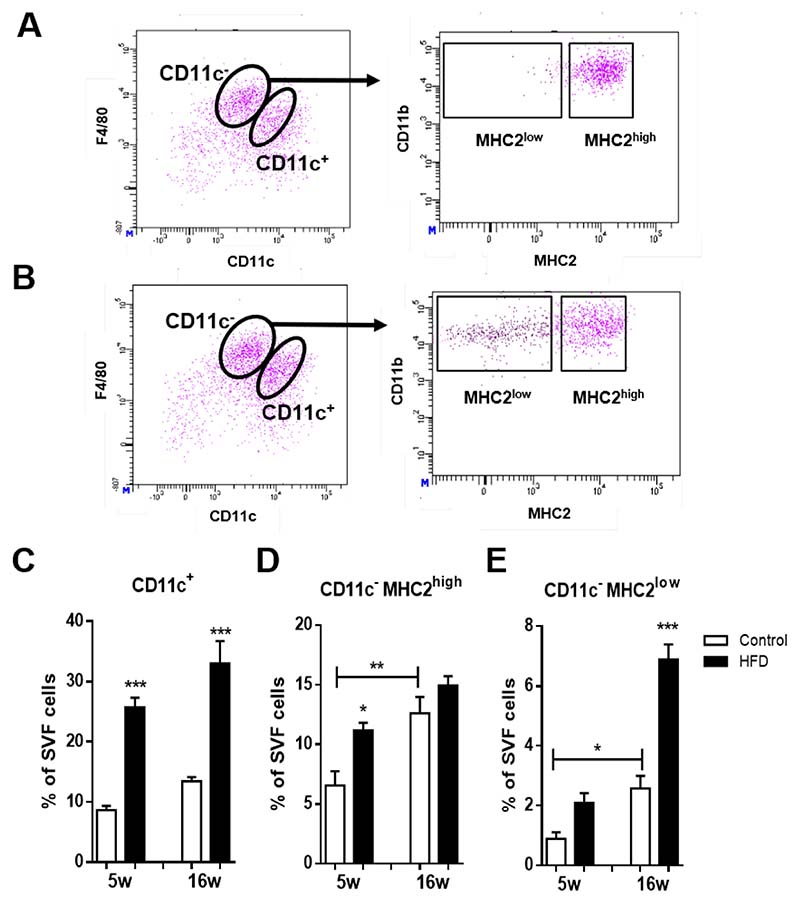
CD11c^−^MHC2^low^ macrophages are a novel and dynamic adipose tissue macrophage population. Flow cytometry analysis of vAT from (**A**) lean and (**B**) obese LDLR^-/-^ mice shows that within CD11c^−^ macrophages, two subsets can be identified based on surface expression of MHC2. Inducing obesity by feeding mice a HFD induces accumulation of (**C**) CD11c^+^ macrophages, (**D**) CD11c^−^MHC2^high^ and (**E**) CD11c^−^MHC2^low^ macrophages in the vAT of LDLR^-/-^ mice (*n* = 7/group). Data is presented as mean ± SEM and analyzed using one-way ANOVA with Tukey’s multiple comparison test. * *p* < 0.05, ** *p* < 0.01, *** *p* < 0.001.

vAT isolated from LDLR^-/-^ mice receiving a HFD for 16 weeks was prepared for flow cytometry and stromal vascular cells were assessed for F4/80, the classical murine macrophage marker and CD11c, which is discriminative for M1 ATMs [15]. F4/80^high^CD11c^−^ cells are considered M2 ATMs. M1 macrophages expressed high levels of MHC2 while M2 ATMs could be further subdivided based on their degree of MHC2 expression into MHC2^high^ and MHC2^low^ M2 ATMs (Figure 1 A,B). The gating strategy to discriminate between the three ATM subsets is shown in Supplemental Figure S1. To assess the phagocytic properties of the CD11c^−^MHC2^low^ ATM subset and confirm the macrophage phenotype of the subsets, we fed LDLR^-/-^ mice a HFD for 10 weeks and the phagocytosing macrophages were depleted two days prior to sacrifice with clodronate liposomes [16]. All three ATM subsets, F4/80^high^CD11c^−^MHC2^low^, F4/80^high^CD11c^−^MHC2^high^, and F4/80^int^CD11c^+^Cd11b^high^ cells were decreased in the vAT of obese LDLR^-/-^ mice whereas the F4/80^−^CD11c^+^CD11b^low^ dendritic cells remained unaffected showing the phagocytic capacity of the three ATM subsets and confirming their macrophage identity (Supplemental Figure S2). F4/80^−^CD11c^+^CD11b^low^ cells have previously been described to possess a dendritic cell signature [17].

As expected, HFD induced an expansion of the CD11c^+^ M1 ATM subset in vAT of LDLR^-/-^ mice (Figure 1C). The CD11c^−^MHC2^high^ M2 ATM subset increased in the vAT after 5 weeks of HFD and remained elevated during prolonged HFD (Figure 1D). The CD11c^−^MHC2^low^ ATM subset increased in the vAT during aging of the mice, however upon HFD, this subset expanded greatly (2.7 fold) (Figure 1E) suggesting that this subset has an important role during obesity. The CD11c^−^MHC2^high^ M2 ATM subtype is also affected by aging as 16 weeks of control diet resulted in increased CD11c^−^MHC2^high^ subset in the vAT which was unaffected by HFD (Figure 1D). Five week high fat feeding also resulted in increased levels of monocytes and dendritic cells in the vAT of LDLR^-/-^ mice whereas 16 weeks of HFD resulted in a decrease of NK and CD4^+^ T-cells (Supplemental Figure S3). Aging also led to an increase of dendritic cells and CD4^+^ T-cells in the vAT on control diet (Supplemental Figure S3). Similar results for immune cell subsets were found in the subcutaneous adipose tissue of LDLR^-/-^ mice after 5 and 16 weeks of HFD (Supplemental Figure S4). Also in the scAT, aging of the LDLR^-/-^ mice resulted in increased levels of CD11c^−^MHC2^high^ ATMs, granulocytes and CD4^+^ T-cells (Supplemental Figure S4). Immune cell subsets in the blood, spleen and bone marrow were not affected by 5 and 16 weeks of HFD (data not shown). In addition, we confirmed the presence of CD11c^−^MHC2^low^ and CD11c^−^MHC2^high^ ATM subsets in the vAT of wild type C57BL/6J mice and observed a similar increase upon a HFD compared as in LDLR^-/-^ mice (data not shown).

### CD11c^−^MHC2^low^ Adipose Tissue Macrophages Are Recruited from the Bone Marrow to the Adipose Tissue during Obesity

**Figure 2:**
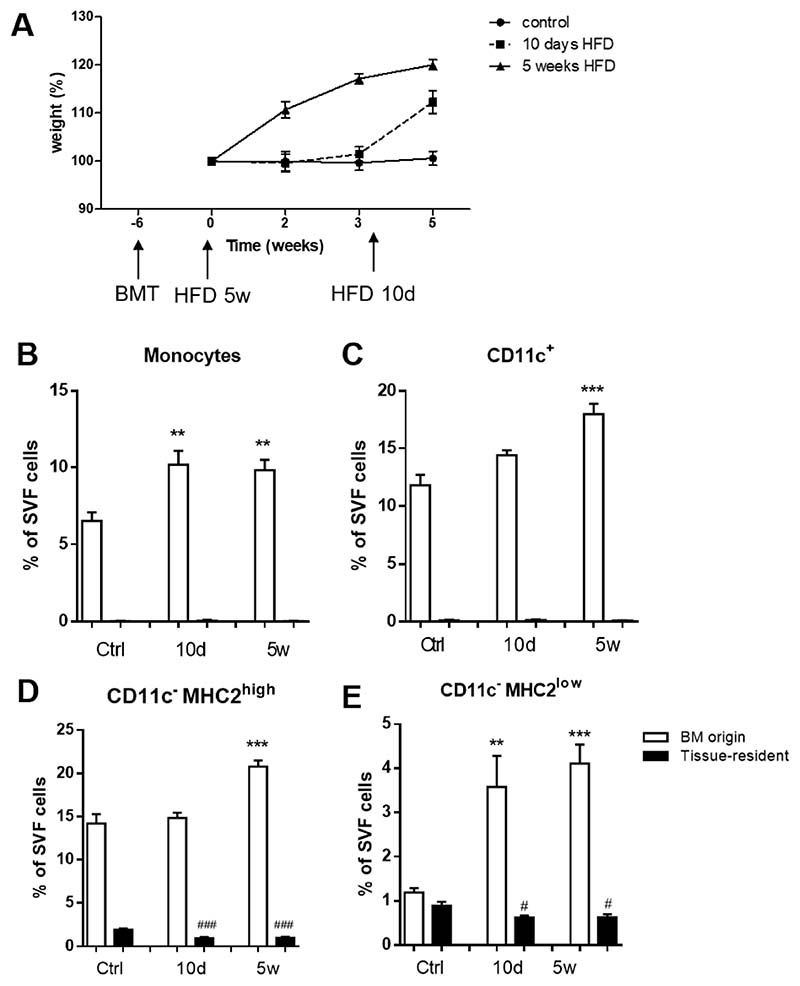
Infiltrating CD11c^−^MHC2^low^ macrophages originate from bone marrow. (**A**) LDLR^-/-^ CD45.2 mice were lethally irradiated and transplanted with LDLR^-/-^ CD45.1 bone marrow. Mice were sacrificed after 10 days (*n* = 6) or 5 weeks (*n* = 7) of HFD. Age-matched animals on a control diet were sacrificed 11 weeks post-transplantation (*n* = 6). vAT was analyzed using flow cytometry. Graph shows setup and body weight during the experiment. White bars represent cells from bone marrow (CD45.1) origin and black bars represent tissue-resident (CD45.2) cells. (**B**) 10 days of high fat feeding results in the accumulation of monocytes from bone marrow origin in vAT and this increase remains stable up to 5 weeks after HFD. (**C**) CD11c^+^ ATMs tended to be increased already after 10 days of HFD, and continued to increase after 5 weeks. (**D**) CD11c^−^ MHC2^high^ ATMs were only increased after 5 weeks of HFD, probably originating from bone marrow. (**E**) CD11c^−^MHC2^low^ ATMs showed comparable distribution of bone marrow versus resident cells. CD11c^−^MHC2^low^ ATMs showed a dramatic increase in numbers as soon as after 10 days of HFD, resulting from bone marrow recruitment. Data is presented as mean ± SEM and analyzed using one-way ANOVA with Dunnett’s Multiple Comparison Test. * significance between BM origin cells due to HFD; ** *p* < 0.01, *** *p* < 0.001, # significance between tissue-resident cells due to HFD; # *p*< 0.05, ### *p* < 0.001.

To investigate tissue residence time and developmental origin of CD11c^−^MHC2^low^ ATMs, CD45.1 bone marrow was transplanted to lethally irradiated CD45.2 LDLR^-/-^ mice, resulting in spontaneous repopulation of the bone marrow. After six weeks of recovery, the transplanted LDLR^-/-^ mice of the 5 weeks HFD group started receiving diet (Figure 2A). Body weight of these mice increased until 120% of their starting weight after 5 weeks HFD. The transplanted LDLR^-/-^ mice receiving 10 days HFD group were on control diet until 10 days before their sacrifice. After switching to a HFD, body weight increased rapidly (Figure 2A). With flow cytometry, ATMs from either acceptor (tissue-resident; CD45.2) or donor (bone marrow; CD45.1) origin were determined.

In steady state, we observed a complete replacement of monocytes and CD11c^+^ macrophages in the vAT of LDLR^-/-^ mice by bone marrow derived cells and virtually no tissue resident monocytes and CD11c^+^ macrophages remained in the vAT (Figure 2B,C). CD11c^−^MHC2^high^ ATMs consisted of almost 89% of cells that were of bone marrow origin, but 11% of the population remained of tissue resident origin (Figure 2D). Strikingly, 43%of the CD11c^−^MHC2^low^ ATMs in the vAT of LDLR^-/-^ mice after lethal irradiation remained of tissue resident origin indicating that this subset of ATMs is either more resistant to irradiation or that there is less cell turnover within this subset (Figure 2E).

Upon 10 days of HFD, monocytes were recruited from the bone marrow to the vAT of LDLR^-/-^ mice and these levels were maintained after 5 weeks of HFD (Figure 2B). The CD11c^+^ ATMs from bone marrow origin accumulated after 5 weeks of HFD in the vAT (Figure 2C), similar as the CD11c^−^MHC2^high^ ATMs (Figure 2D). The CD11c^−^MHC2^low^ ATM subset was rapidly increased after 10 days of HFD and remained elevated after 5 weeks of HFD as a result of bone marrow recruitment (Figure 2E).

### CD11c^−^MHC2^low^ Adipose Tissue Macrophages Adopt an Inflammatory Phenotype during Obesity

**Figure 3:**
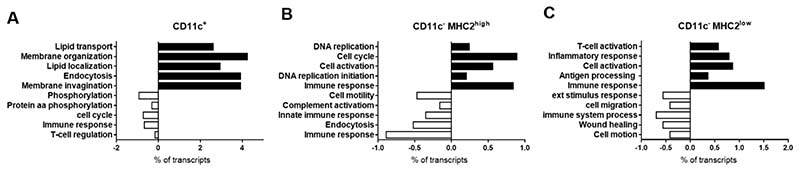
Microarray of the CD11c^+^, CD11c^−^MHC2^high^ and CD11c^−^MHC2^low^ ATMs. Using fluorescent activated cell sorting, the ATM subsets were isolated from lean (3 pooled samples from *n* = 6 LDLR^-/-^ mice/sample) and obese animals (3 pooled samples from *n* = 4 LDLR^-/-^ mice/sample). Genes regulated by high fat feeding in the three ATM subsets were analyzed. GO annotation analysis showed diet-induced effect in (**A**) CD11c^+^, (**B**) CD11c^−^MHC2^high^, and (**C**) CD11c^−^MHC2^low^ ATMs.

**Figure 4:**
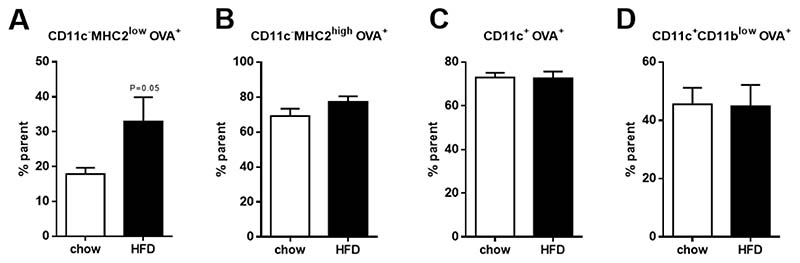
Antigen uptake and processing in the three subsets of ATMs and dendritic cells. Uptake and processing of OVA in (**A**) CD11c^−^MHC2^low^ ATMs, (**B**) CD11c^−^MHC2^high^ ATMs, (**C**) CD11c^+^ ATMs and (**D**) CD11c^+^CD11b^low^dendritic cells in the vAT of LDLR^-/-^ mice after 5 weeks of HFD feeding (*n* = 4/group). Data is presented as mean ± SEM and analyzed using Mann-Whitney *t*-test.

To determine the differentially expressed genes in CD11c^+^, CD11c^−^MHC2^low^ and CD11c^−^MHC2^high^ ATMs, we performed microarray expression analysis on FACS-sorted cells of these subsets. In steady-state, in LDLR^-/-^ mice on the control diet, CD11c^−^MHC2^high^ ATMs express 1528 genes higher and 1152 genes lower compared to CD11c^+^ ATMs (adjusted *p*-value < 0.05). This shows the great variety between the CD11c^+^ M1 and CD11c^−^ M2 ATM subsets. However, comparing CD11c^−^MHC2^high^ ATMs to CD11c^−^MHC2^low^ ATMs, only 35 genes were expressed higher and 88 genes were expressed lower. This indicates that in basal conditions the two M2 subsets are much alike. Upon a HFD given to LDLR^-/-^ mice, gene expression profile of all three ATM subsets change; CD11c^+^ M1 ATMs paradoxically gain a slightly attenuated inflammatory phenotype as they upregulate 667 genes from pathways involved in lipid transport, membrane organization and endocytosis, and downregulate 334 genes from pathways linked to phosphorylation and immune response (Figure 3A). CD11c^−^MHC2^high^ ATMs showed upregulation of 261 genes from pathways involved in DNA replication, cell cycle and cell activation (Figure 3B). CD11c^−^MHC2^low^ ATMs seemed to exhibit highly distinct characteristics as they upregulated 273 genes involved in immune response and immune cell activation pathways (Figure 3C), the latter including a cluster of genes involved in cell mobility. Genes upregulated for the cluster cell mobility included CCL2, CCR2, CCR5 and CXCR4 (Supplemental Table S2). Genes upregulated in the cluster immune response included several genes involving the transcription factor CiiTA and histocompatibility 2 target genes (Supplemental Table S3). Since this upregulation of genes also involved antigen presentation related genes, we determined whether there is a difference in antigen uptake and processing capacity, an inflammatory characteristic of macrophages. Therefore, we explored the ability of the three ATM subsets and dendritic cells to take up and process labeled ovalbumin (DQ-OVA) after HFD, as a measure for antigen processing. The results show that only the CD11c^−^MHC2^low^ ATM subset increases the uptake and processing of OVA during 5 weeks of HFD feeding (Figure 4A), whereas high fat feeding did not affect antigen processing capacity of CD11c^−^MHC2^high^ ATMs, CD11c^+^ ATMs and dendritic cells (Figure 4B–D). The increased antigen processing capacity of the CD11c^−^MHC2^low^ ATMs could suggest that T-cell activation is increased in the adipose tissue of LDLR^-/-^ mice upon HFD. However, the amount of CD4^+^ T-cells in the vAT and scAT upon 16 weeks of HFD are decreased (Supplemental Figures S3 and S4), arguing against a local activation of T-cells by these macrophages. To assess whether there were changes in the T-cell cytokine profile in the blood, we measured multiple cytokines in plasma of these mice after 5 and 16 weeks of HFD. The results showed that after 5 and 16 weeks of HFD, plasma IL-10 levels were increased (data not shown). However, the other cytokines (IFNγ, IL-12, IL-2, IL-4, and IL-5), reflective of either Th1 or Th2 activation, were not affected by the HFD (data not shown). Together, these data do not support T-cell specific changes in the plasma and vAT after HFD coinciding with an accumulation of CD11c^−^MHC2^low^ ATMs.

### The CD11c^−^MHC2^low^ Phenotype of ATMs Can Be Induced by PA and OA

**Figure 5:**
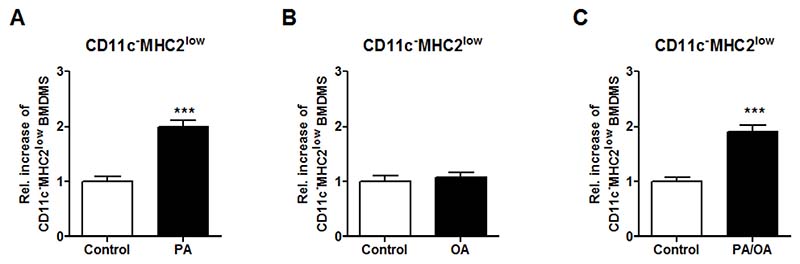
Free fatty acid PA induce the CD11c^−^MHC2^low^ phenotype in vitro. BMDMs were stimulated in vitro with 50 μM PA, 50 μM OA and a combination of 50 μM PA and OA to induce macrophage phenotype. Flow cytometry analysis of the stimulated BMDMs to determine the induction of the CD11c^−^MHC2^low^ subset in the BMDMs after (**A**) PA, (**B**) OA and (**C**) PA and OA stimulation. Representative data from 3 experiments with *n* = 3–4/experiment. Data is presented as mean ± SEM and analyzed using an unpaired Student’s *t*-test. *** *p* < 0.001.

A key characteristic of obesity is the presence of elevated levels of free fatty acids (FFAs) in the adipose tissue. These FFAs include, among others, dietary fatty acids such as PA and OA which are known to have pro- and anti-inflammatory effects respectively. Previously it was revealed that lipolysis in mouse adipose tissue regulates the accumulation of macrophages in the adipose tissue [18]. Since PA and OA are the major FFA types in lard, we tested their effects on the phenotype of macrophages. BMDMs were stimulated in vitro with PA, OA and a mix of PA and OA followed by flow cytometry to determine the BMDM phenotype. The stimulation with PA and the combination of PA and OA induced the CD11c^−^MHC2^low^ macrophages in vitro (Figure 5A,C), whereas this was not found for OA stimulation alone (Figure 5B). Our data indicate that the microenvironment of the macrophage is crucial for its phenotype and functional characteristics during obesity.

## Discussion

Our study reveals that the alternative M2 macrophage subset in adipose tissue of LDLR^-/-^ mice can be subdivided into two separate subsets based on their MHC2 expression: CD11c^−^MHC2^high^ and CD11c^−^MHC2^low^ macrophages. Canonically, ATMs, like all macrophages, are subdivided in two distinct populations: inflammatory M1 macrophages and anti-inflammatory or resolving M2 macrophages [19]. However, the validity of this subdivision has been called into question in many recent studies [20,21]. In fact, recent studies report many different subtypes of ATMs [22–25], each of which has their own unique characteristics. We investigated the ATM subsets in the adipose tissue during obesity in LDLR^-/-^ mice. Our results show that in the adipose tissue of lean mice, the gene expression profiles of CD11c^−^MHC2^high^ and CD11c^−^MHC2^low^ macrophages were very similar, indicating comparable functions. However, upon HFD the phenotype of the CD11c^−^MHC2^low^ macrophages shifted to a more inflammatory phenotype suggesting that this subset contributes to adipose tissue inflammation during obesity. Surprisingly, genes involved in antigen presentation were induced by HFD feeding in CD11c^−^MHC2^low^ ATMs. It should be noted that the CD11c^−^MHC2^low^ subset comprises 6–7% of the stromal vascular fraction whereas the CD11c^−^MHC2^high^ and CD11c^+^ subset represent 15% and 35% respectively. Therefore, on one hand, the changes in gene expression profile of the CD11c^−^MHC2^low^ subset will have a smaller impact due to their low percentage in the vAT as compared to the other subsets. On the other hand, small transcriptional changes in the CD11c^+^ ATM subset could have bigger effects since they are more abundant in the vAT.

We showed that the CD11c^−^MHC2^low^ ATM subset was able to phagocytose as we were able to deplete them using clodronate liposomes. Clodronate liposomes are known to selectively deplete phagocytosing macrophages from organs and tissue [16,26], confirming that this is indeed a macrophage subset. To confirm whether the CD11c^−^MHC2^low^ subset is more potent in antigen processing, we determined the uptake and processing of DQ-OVA as a measure of antigen processing capacity. Our results revealed that during obesity, the ability to take up and process DQ-OVA was increased in CD11c^−^MHC2^low^ ATMs but was not changed in the CD11c^−^MHC2^high^ ATMs, CD11c^+^ ATMs and the dendritic cells. Together with the unaltered cytokine profile in the plasma, our data do not support T-cell specific changes in the plasma and vAT after HFD. A previous study by Morris et al. showed that obesity in mice resulted in an increase in MHC2 levels, which was more prominent on CD11c^+^ compared to CD11c^−^ ATMs. However, it should be noted that in this study CD11c^−^ ATMs were not subdivided based on MHC2 surface expression, making a direct comparison with our data impossible. Moreover, in contrast with our data, Morris et al. found an increase of T-cell co-stimulatory molecules on ATMs in vAT [27]. Yet, it remains unclear whether self-antigens are derived from the obese adipose. In turn, Porsche et al. revealed that dendritic cells in the adipose tissue are more potent activators of CD4^+^ T-cells compared to ATMs and that blocking CD4^+^ T-cell activation in AT by reducing MHC2 on dendritic cells does not affect chronic low grade inflammation in adipose tissue with obesity [28]. Together, these results suggest that ATMs induce inflammation in the obese adipose tissue independent of their capacity to present antigen to T-cells.

The CD11c^−^MHC2^low^ subset of ATMs may have been be overlooked by previous researchers investigating ATM subsets. Rajbhandari et al. performed single cell sequencing on the SVF isolated from murine adipose tissue and revealed three subsets of macrophages after clustering analysis [29]. Although they do not go more into detail on these three macrophage subsets, it could be plausible that it divides the CD11c^+^, CD11c^−^MHC2^high^ and CD11c^−^MHC2^low^ subsets into three unique clusters. Weinstock et al. also performed single cell sequencing on CD45^+^ immune cells isolated from the vAT from lean, obese and calorie restricted mice. Data analysis revealed that over 50% of the immune cells in the vAT are macrophages and they could be clustered into 7 distinct clusters with differences in metabolic and inflammatory functions [30], revealing a great heterogeneity in macrophage subsets in the vAT. In addition to singe cell sequencing data, it has been reported that gating strategies based on CD11c expression may cause relevant subsets to be overlooked. Hill et al. report the existence of CD9^+^ and Ly6c^+^ ATMs which form functionally distinct ATM subsets, but within these subsets, there is a highly heterogeneous distribution of CD11c expression [24]. In our microarray dataset, we find 3.34 fold higher CD9 expression in the CD11c^+^ ATM subset compared to the CD11c^−^MHC2^high^ ATM subset, whereas Ly6c is equal between both subsets. When comparing CD11c^−^MHC2^high^ with CD11c^−^MHC2^low^ ATMs, we find 2.1 fold higher expression of CD9 in CD11c^−^MHC2^low^ ATMs. This increase in CD9 expression together with the inflammatory phenotype suggests that the CD11c^−^MHC2^low^ ATMs starts resembling the CD11c^+^ ATM subset in HFD conditions. Silva et al. identified 4 distinct ATM subsets in steady state and after chronic HFD, though unfortunately they also did not observe a CD11c^−^MHC2^low^ ATM subset [31]. These data by other groups illustrate the difficulty of establishing gating strategies that accurately recapitulate ATM subsets in vivo and that even unbiased methods, like single cell sequencing do not provide definitive answers with respect to existing ATM populations.

There has been some debate on whether ATMs expand in the adipose tissue due to local proliferation or whether they are solely recruited from the bone marrow. We have determined the origin of this novel ATM subset by making use of a bone marrow transplantation strategy in which CD45.1 donor BM cells were injected into lethally irradiated CD45.2 acceptor mice. CD45.1 donor BM cells will spontaneously repopulate the bone marrow of the irradiated acceptor mice. Tissue resident inflammatory cells will be CD45.2 allowing to discriminate between bone marrow-derived and tissue resident immune cells. The results of the bone marrow transplantation experiment show that, in the steady state, monocytes and CD11c^+^ ATMs in vAT are rapidly replaced by cells from the bone marrow. However, half of the CD11c^−^MHC2^low^ ATMs remain of CD45.2 origin, suggesting that there is slower cell turnover within this subset. Alternatively, we cannot exclude that these cells are more radioresistant than the other populations. Upon HFD, there is a massive increase in bone marrow recruited ATMs in the vAT. This increase in bone marrow origin ATMs was seen after five weeks of HFD and for the three subsets. Interestingly, the CD11c^−^MHC2^low^ subset rapidly accumulated in the adipose tissue after 10 days of HFD and remained elevated after 5 weeks HFD. We also revealed that this subset increases even further with prolonged HFD (16 weeks). In our study, it is difficult to determine the location of the ATM subsets in the adipose tissue, which would also be of great importance concerning their function in the tissue. Based on the microarray data, we could not find a marker specific enough to distinguish the CD11c^−^MHC2^low^ subset from the other ATM subset (CD11c^−^MHC2^high^) using immunohistochemistry. Relying on the absence of CD11c, presence of f4/80 and reduced MHC2 expression makes it difficult to separate these two subsets during immunohistochemical staining of adipose tissue. Future studies using reporter mice or knock-out models may resolve this issue. Our data confirms previous work of Weisberg et al. which demonstrated that the accumulation of ATMs in the obese adipose tissue was due to an increased influx of bone marrow-derived precursors [32]. Nevertheless, we cannot exclude the possibility of local proliferation of ATMs after recruitment from the bone marrow as it has been reported that local proliferation of ATMs during obesity contributes to the accumulation of ATMs [33,34]. Our data show that CD11c^−^MHC2^high^ ATMs, although their accumulation seemed to originate from bone marrow precursors, displayed upregulation of genes involved in DNA replication and cell cycle implicating a proliferative phenotype of the recruited cells. Although Amano et al. did not observe differences in proliferation rates between CD11c^+^ and CD11c^−^ ATM subsets, our data are in line with the results of Haase et al, showing that proliferating cells preferentially showed M2 polarization. However, since we did not determine local proliferation by Ki67 staining and EdU or BrdU incorporation these transcriptomic results should be interpreted with care. Moreover, it could be speculated that the irradiation treatment, due to cell death, triggers monocyte influx from the bone marrow upon HFD which will normally not be induced by the HFD. It is known that circulating monocytes will fill the empty macrophage niche in the tissues after depletion [35,36]. Future experiments using shielding of the adipose tissue during irradiation, preserving tissue-resident ATMs in combination with tracer studies may resolve these pitfalls. Nevertheless, our experiments do suggest differences in the tissue residence time and recruitment of the different macrophage subsets.

The appearance of the CD11c^−^MHC2^low^ ATM subset in obese vAT raises the question which factors drive the MHC2^low^ phenotype during obesity. FFAs are known to be increased during obesity. In human adipose tissue, OA is the most abundant FFA, whereas PA is the second most abundant [10]. Moreover, PA and OA are the major FFA types in lard, which was the main component of the HFD used in our in vivo experiments. Therefore, we selected PA and OA to investigate the potential to induce the CD11c^−^MHC2^low^ phenotype in BMDMs. Both FFAs are documented to have an effect on macrophages; PA has inflammatory effects [37] whereas OA was able to induce arginase 1 expression, a hallmark of M2 activation, in RAW 264.7 macrophages in vitro [38]. We found that PA and the combination of PA and OA was able to induce the CD11c^−^MHC2^low^ phenotype in BMDMs. FFAs were previously shown to regulate the macrophage content of the adipose tissue. Kostelli et al. revealed that increased lipolysis (release of FFA) in the adipose tissue, which also occurs during weight loss, results in increased CD11c^−^ ATMs in the adipose tissue [18]. Future studies are needed to investigate whether or not this increase in CD11c^−^ ATMs represents accumulation of CD11c^−^MHC2^low^ ATMs.

## Conclusions

Our data shows that CD11c^−^ M2 ATMs in mouse adipose tissue consist of two separate populations with its own specific functions. These CD11c^−^ ATMs can be subdivided into two distinct subsets: CD11c^−^MHC2^high^ and CD11c^−^MHC2^low^. Microarray data shows that both subsets have a different phenotype with CD11c^−^MHC2^low^ ATMs having a more pro-inflammatory phenotype compared to the classic M2 phenotype attributed to CD11c^−^ ATMs. We reveal that ATMs are recruited from the bone marrow after lethal irradiation. Yet, CD11c^−^MHC2^low^ ATMs also remain in the tissue after irradiation, suggesting less cell turnover. Moreover, our data shows that FFAs PA is able to induce the CD11c^−^MHC2^low^ phenotype in vitro in BMDMs, suggesting that the microenvironment of the adipose tissue contributes to the phenotype of the ATMs. This subset of ATMs increases in the adipose tissue during inflammation and could contribute to inflammatory-related diseases such as diabetes. Our data indicates that previous research on M2 macrophages in the murine adipose tissue disregarded the inflammatory function of the CD11c^−^MHC2^low^ ATMs which could influence research results. In addition, future research is needed to elucidate the contribution of this specific subset to obesity-related diseases and for translation into the human situation.

## Supplementary Material

The supplementary material is available online at https://doi.org/10.20900/immunometab20200014.

Supplementary file

## Data Availability

**Data Availability** The dataset of the study is available from the authors upon reasonable request.

## Abbreviations

ATMs: adipose tissue macrophages
BMDMs: bone marrow-derived macrophages
LCM: L929 cell conditioned medium
HFD: high fat diet
vAT: visceral adipose tissue
SVF: stromal vascular fraction
OVA: ovalbumin
T2D: type 2 diabetes
FFAs: free fatty acids
PA: palmitate
OA: oleate
LDLR: low-density lipoprotein receptor
