# Supplementary material for: CD11c−MHC2low Macrophages Are a New Inflammatory and Dynamic Subset in Murine Adipose Tissue
Source: Immunometabolism. Author manuscript; Available in PMC 2022 Jan 4. (PMC7612162; doi:10.20900/immunometab20200015)
Supplement: Supplementary file [file EMS121179-supplement-Supplementary_file.pdf]

Supplementary Material

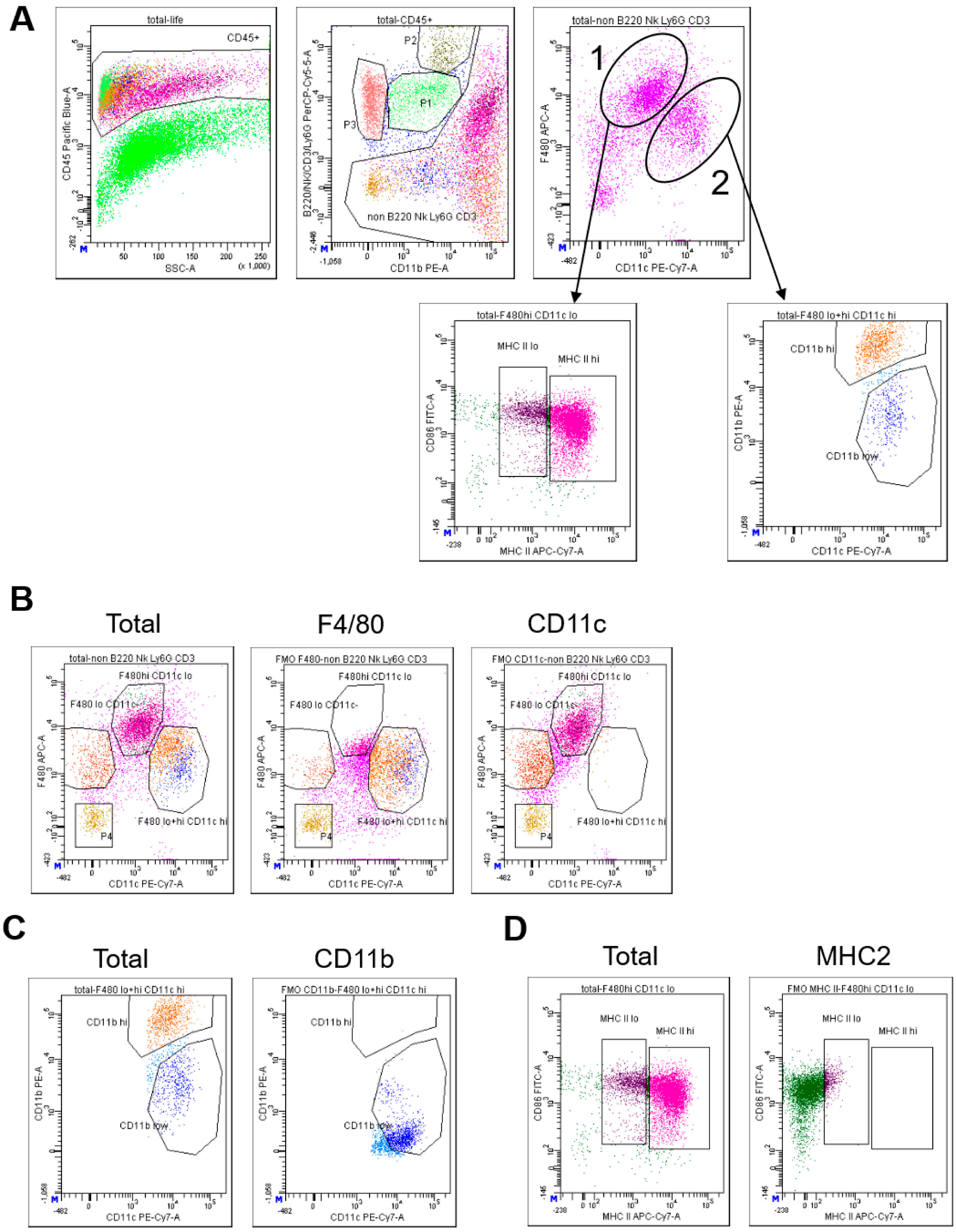

Supplemental Figure S1. Gating strategy of vAT flow cytometry analysis. (**A**) The gating strategy of the flow cytometry analysis of vAT. First, immune cells are selected based on CD45 and side scatter (SSC). From this subset, B-cells, NK-cells, T-cells and granulocytes are removed using B220, NK, CD3 and Ly6G. Cells negative for this cocktail are selected and used to determine F4/80 and CD11c expression. The cells represented in gate 1 are F4/80^{+}CD11c^{-} M2 macrophages and can be subdivided into F4/80^{+}CD11c^{-}MHC^{high} and F4/80^{+}CD11c^{-}MHC^{low}. The cells represented in gate 2 are F4/80^{low}CD11c^{+} and can be divided into F4/80^{low}CD11c^{+}CD11b^{+} M1 macrophages and F4/80^{low}CD11c^{+}CD11b^{-} dendritic cells. FMO of the F4/80, CD11c (**B**), CD11b (**C**) and MHC2 (**D**) staining.

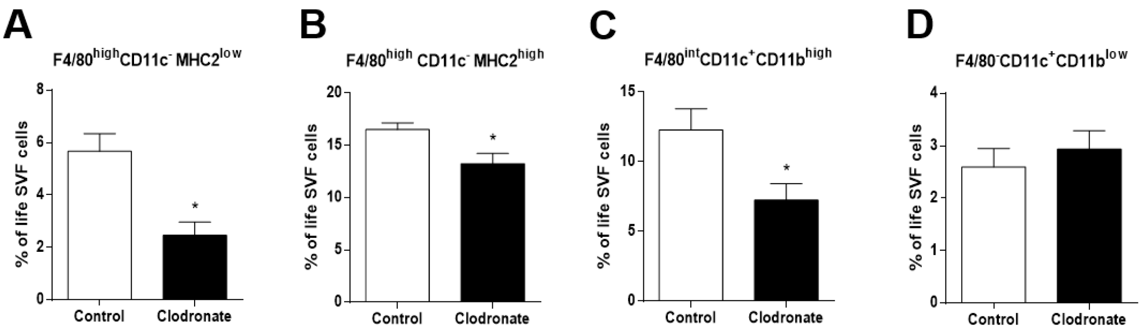

Supplemental Figure S2. F4/80^{high}CD11c^{-} MHC2^{low} macrophages are bona fide phagocytosing macrophages. LDLR$^{-/-}$ mice were fed a HFD and two days prior to sacrifice, clodronate liposomes are injected to deplete macrophages (n = 3/group). Uptake of the clodronate liposomes results in depletion of F4/80^{high}CD11c^{-}MHC2^{low} (A), F4/80^{high}CD11c^{-}MHC2^{high} (B) and F4/80^{int}CD11c^{+}CD11b^{high} (C) ATMs whereas the dendritic cells (F4/80^{-}CD11c^{+}CD11b^{low} (D) remain unaffected showing that F4/80^{high}CD11c^{-} MHC2^{low} cells are indeed phagocytosing macrophages. Data is presented as mean ± SEM and analyzed using unpaired students t-test. * $p<0.05$

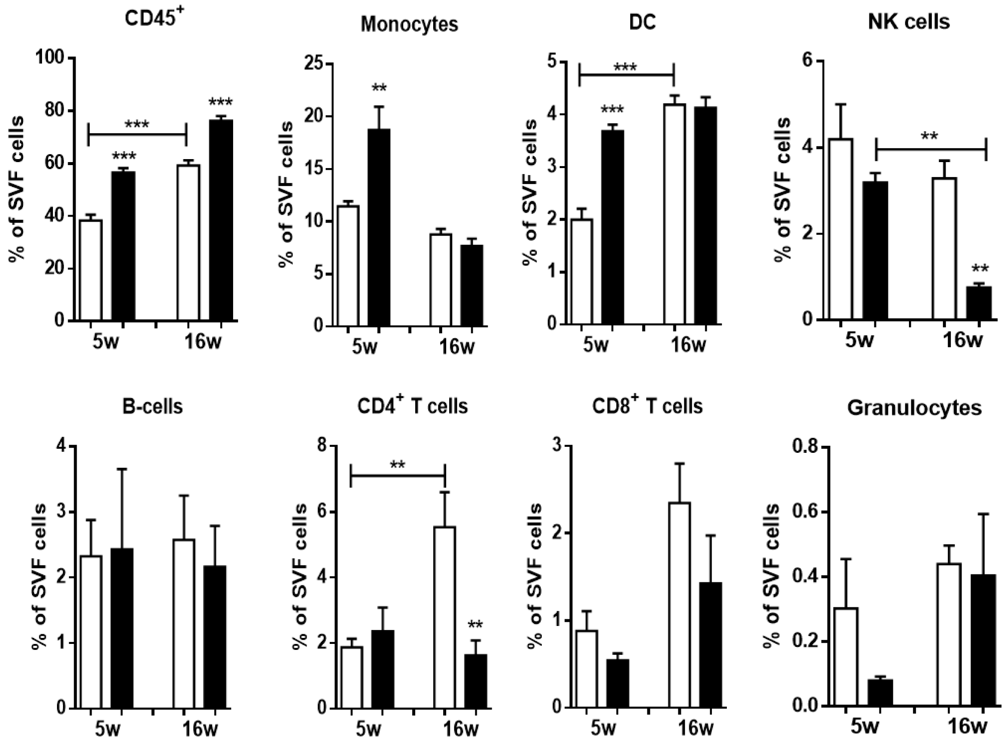

Supplemental Figure S3. HFD induced changes in the immune cell populations in the vAT. LDLR$^{-/-}$ mice were fed a HFD or control diet for either 5 weeks or 16 weeks (n = 7/group). Flow cytometry was used to determine the various immune cell subsets in the vAT: CD45^{+} immune cells, monocytes, dendritic cells (DC), NK cells, B-cell, CD4^{+} T-cells, CD8^{+} T-cells and granulocytes. Data is presented as mean ± SEM and analyzed using one-way ANOVA and Tukey's multiple comparison test. ** $p<0.01$, ***$p<0.001$

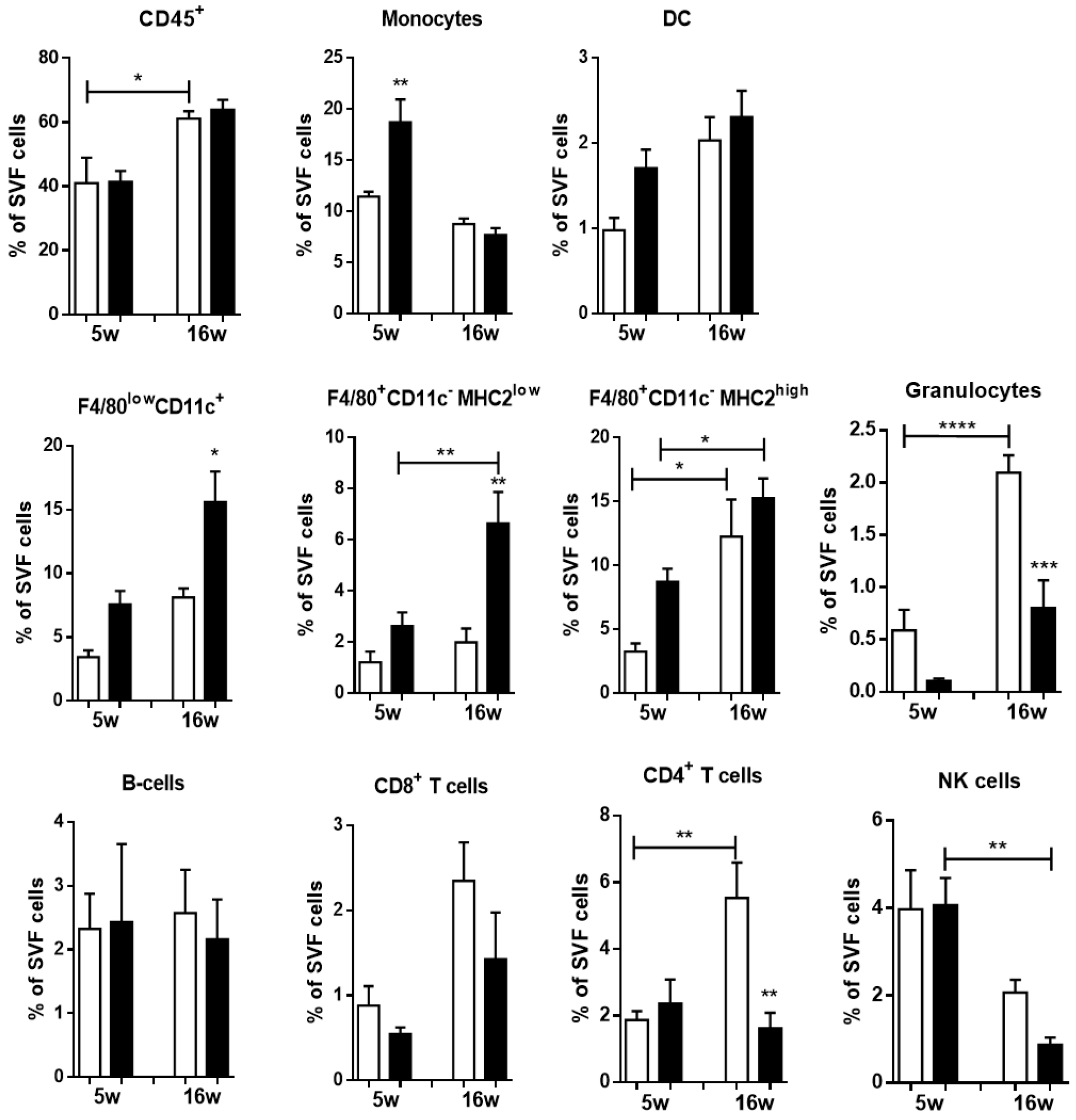

Supplemental Figure S4. HFD induced changes in the immune cell populations in the subcutaneous (sc)AT. LDLR$^{-/-}$ mice were fed a HFD or control diet for either 5 weeks or 16 weeks (n = 7/group). Flow cytometry was used to determine the various immune cell subsets in the scAT: CD45^{+} immune cells, monocytes, dendritic cells (DC), F4/80lowCD11c+ M1 macrophages, F4/80^{+}CD11c^{-}MHC2^{low} M2 macrophage subset, F4/80^{+}CD11c^{-}MHC2^{high} M2 macrophage subset, granulocytes, B-cell, CD8^{+} T-cells, CD4^{+} T-cells and NK cells. Data is presented as mean ± SEM and analyzed using one-way ANOVA and Tukey's multiple comparison test. *p<0.05, ** p<0.01, ***p<0.001, ****p<0.0001

Supplemental Table S1. The total amount of cells sorted from the vAT of chow (sample 1 – 3) and HFD (sample 4 – 6) fed LDLR$^{-/-}$ mice for RNA extraction. Sample 1 – 3 are a pool of n=6 LDLR$^{-/-}$ mice/sample and sample 4 – 6 are a pool of n=4 LDLR$^{-/-}$ mice/sample. After sorting, these samples are used for RNA extraction and microarray analysis.

	**CD11c^{+}**	**CD11c^{-} MHC2^{low}**	**CD11c^{-} MHC2^{high}**
Sample 1	80.138	94.041	218.870
Sample 2	52.989	68.183	138.003
Sample 3	97.592	129.999	403.988
Sample 4	609.583	218.746	274.308
Sample 5	671.260	271.292	302.414
Sample 6	567.515	151.871	268.821

Supplemental Table S2. Microarray data from the cluster mobility of CD11c^{+}, CD11c^{-}MHC2^{low} and CD11c^{-}MHC2^{high} ATM sorted from vAT of LDLR$^{-/-}$ mice after HFD. Microarray data showed the fold change and adjusted p-value of the various genes represented in the cluster mobility.

		CD11c^{+}		CD11c^{-}MHC2^{low}		CD11c^{-}MHC2^{high}	
Gene	Description	Fold change	Adjusted p-value	Fold change	Adjusted p-value	Fold change	Adjusted p-value
Ccl4	chemokine (C-C motif) ligand 4	1.033614	0.952041	2.65275	0.00318	1.754554	0.090675
Ccr2	chemokine (C-C motif) receptor 2	-1.44551	0.062303	2.332817	0.000306	1.39017	0.161369
Ccr5	chemokine (C-C motif) receptor 5	1.090284	0.806116	1.705698	0.044073	1.307065	0.431451
Cxcr4	chemokine (C-X-C motif) receptor 4	1.536505	0.107202	1.881588	0.035412	1.437431	0.296844
Pdgfb	platelet derived growth factor, B polypeptide	2.133762	0.020665	2.433474	0.016557	1.749664	0.154704
Myo1f	myosin IF	1.44276	0.039203	1.65258	0.011785	1.376465	0.12071
Tgfbr1	transforming growth factor, beta receptor I	2.651582	0.001036	1.935949	0.043951	1.712958	0.094582

Supplemental Table S3. Microarray data from the cluster immune response of $CD11c^{+}$, $CD11c^{-}MHC2^{low}$ and $CD11c^{-}MHC2^{high}$ ATM sorted from vAT of $LDLR^{-/-}$ mice after HFD. Microarray data showed the fold change and adjusted p-value of the various genes represented in the cluster immune response.

		$CD11c^{+}$		$CD11c^{-}MHC2^{low}$		$CD11c^{-}MHC2^{high}$	
Gene	Description	Fold change	Adjusted p-value	Fold change	Adjusted p-value	Fold change	Adjusted p-value
H2-Aa	histocompatibility 2, class II antigen A, alpha	-1.03836	0.907799	1.793378	0.005541	-1.00491	0.996585
H2-Ab1	histocompatibility 2, class II antigen A, beta 1	-1.10103	0.713864	1.773606	0.006394	1.005548	0.995649
H2-DMa	histocompatibility 2, class II, locus DMa	-1.2316	0.437951	1.94865	0.009922	-1.03947	0.968386
H2-DMb1	histocompatibility 2, class II, locus Mb1	-1.1262	0.764763	2.009455	0.021756	1.052778	0.962456
H2-Eb1	histocompatibility 2, class II antigen E beta	-1.07124	0.868594	2.494813	0.001416	-1.0093	0.994811
Ciita	class II transactivator	-1.23128	0.518934	2.025003	0.018145	-1.04206	0.971891
Fcgr4	Fc receptor, IgG, low affinity IV	1.998848	0.143996	3.175419	0.027762	2.468424	0.082999
